# Platelet Recovery and Mortality in Septic Patients with Thrombocytopenia: A Propensity Score-Matched Analysis of the MIMIC-IV Database

**DOI:** 10.3390/jcm15020884

**Published:** 2026-01-21

**Authors:** Yi Zhou, Xiangtao Zheng, Yanjun Zheng, Zhitao Yang

**Affiliations:** Department of Emergency Medicine, Ruijin Hospital, Shanghai Jiao Tong University School of Medicine, Shanghai 200025, China; zy12442@rjh.com.cn (Y.Z.); zxt01p01@rjh.com.cn (X.Z.); zyj12378@rjh.com.cn (Y.Z.)

**Keywords:** sepsis, thrombocytopenia, platelet recovery, ICU mortality, MIMIC-IV, prognosis

## Abstract

**Background:** Thrombocytopenia (platelet count < 100 × 10^9^/L) occurs in 20–40% of critically ill patients with sepsis and is associated with adverse outcomes. Most prior studies have treated thrombocytopenia as a static risk indicator rather than a dynamic process. We investigated whether platelet recovery within 7 days provides independent prognostic information in patients with sepsis. **Methods:** We performed a retrospective cohort study using the MIMIC-IV database. Among 22,513 adults with sepsis admitted to intensive care units, 5401 developed thrombocytopenia within 24 h of admission and had sufficient follow-up data. The primary exposure was sustained platelet recovery to ≥100 × 10^9^/L within 7 days. The primary outcomes were 28-day and in-hospital mortality. Propensity-score matching and overlap weighting were used to adjust for demographic characteristics, comorbid conditions, illness severity, and organ-support therapies. **Results:** Among 5401 septic ICU patients with thrombocytopenia, 3193 (59%) achieved platelet recovery within 7 days. A total of 2056 patients (38%) recovered by day 3, and 1137 (21%) recovered between days 4 and 7. After multivariable adjustment, platelet recovery was independently associated with markedly lower mortality (adjusted risk ratio, 0.56; 95% CI, 0.53–0.67 for in-hospital death; and 0.60; 95% CI, 0.53–0.67 for 28-day death) and more than a doubling of survival time (adjusted ratio, 2.08; 95% CI, 1.65–2.63). Early and intermediate recovery conferred similar benefits. Higher baseline platelet counts, antiplatelet therapy, and heparin use were associated with recovery, whereas cirrhosis, greater illness severity, and continuous renal replacement therapy were associated with non-recovery. **Conclusions:** In patients with sepsis and thrombocytopenia, platelet recovery within 7 days was a strong and independent predictor of survival. Exploratory timing-stratified analyses yielded similar associations across subgroups. These findings support platelet recovery as a useful prognostic marker reflecting broader physiologic stabilization in sepsis.

## 1. Introduction

Sepsis is a leading cause of morbidity and mortality in intensive care units worldwide, affecting millions of patients annually and imposing a substantial global healthcare burden [1,2]. Thrombocytopenia in ICU patients, commonly defined as a platelet count below 100 × 10^9^/L, occurs in approximately 20–40% of critically ill patients with sepsis [3,4] and has long been recognized as a marker of disease severity [5]. Lower platelet counts at presentation have been consistently associated with higher mortality and greater degrees of organ dysfunction in large observational cohorts [6,7]. Beyond their role in hemostasis, platelets are essential for immune regulation, endothelial integrity, and microvascular perfusion [8,9]. Platelet depletion in sepsis reflects the interplay among increased consumption, immune-mediated clearance, impaired thrombopoiesis, and sequestration in injured tissues—hallmarks of sepsis-associated coagulopathy and multiorgan dysfunction [10,11]. Thrombocytopenia in critically ill patients may result from multiple mechanisms, including decreased bone marrow production, increased peripheral destruction, splenic sequestration, and dilution [12,13]. In the context of sepsis, these mechanisms are often interrelated, reflecting the complex interplay between infection, inflammation, and coagulopathy.

Despite this well-established association, most prior investigations have focused on the presence or nadir of thrombocytopenia as a static risk marker [5]. These studies have typically evaluated platelet counts at ICU admission or at a single point during critical illness [14], an approach that fails to capture the dynamic trajectory of platelet recovery across the course of hospitalization [7]. Consequently, it remains unclear whether thrombocytopenia merely reflects baseline severity or whether platelet recovery itself carries independent prognostic significance [15,16]. Few studies have systematically characterized platelet recovery patterns [17], and even fewer have examined the clinical implications of recovery timing (≤3 days vs. 4–7 days) or different recovery trajectories [6].

The Medical Information Mart for Intensive Care (MIMIC-IV) database provides an opportunity to address these knowledge gaps [18]. Encompassing de-identified, high-resolution electronic health records from a U.S. academic center over more than a decade, it includes detailed laboratory measurements, vital signs, therapeutic interventions, and clinical outcomes. Its longitudinal structure and open-access format make it well-suited to characterize platelet trajectories from the onset of thrombocytopenia and to evaluate their associations with clinical outcomes in septic ICU populations.

In this retrospective cohort study using MIMIC-IV, we evaluated whether sustained platelet recovery (to ≥100 × 10^9^/L) within 7 days of thrombocytopenia onset is independently associated with clinical outcomes in septic patients. Secondary objectives included evaluating whether the timing of recovery (≤3 days vs. 4–7 days) influences prognosis and identifying baseline or treatment-related factors associated with recovery. By examining dynamic recovery processes rather than static thrombocytopenia alone, we sought to clarify the prognostic relevance of platelet restoration in sepsis and to inform risk stratification in critical care.

## 2. Methods

### 2.1. Study Design and Data Source

We conducted a retrospective cohort study using the Medical Information Mart for Intensive Care IV (MIMIC-IV, version 2.0) database hosted on PhysioNet (certification number: 63414301). The database contains de-identified electronic health records from patients admitted to the intensive care units of Beth Israel Deaconess Medical Center between 2008 and 2019 [18]. The project was approved by the institutional review boards of the Massachusetts Institute of Technology and Beth Israel Deaconess Medical Center, with a waiver of informed consent. One investigator (Yi Zhou) completed the required data-use training, obtained database access credentials, and oversaw data extraction and verification. All analyses were performed using a fixed dataset extracted in June 2024.

### 2.2. Study Population

**Figure 1:**
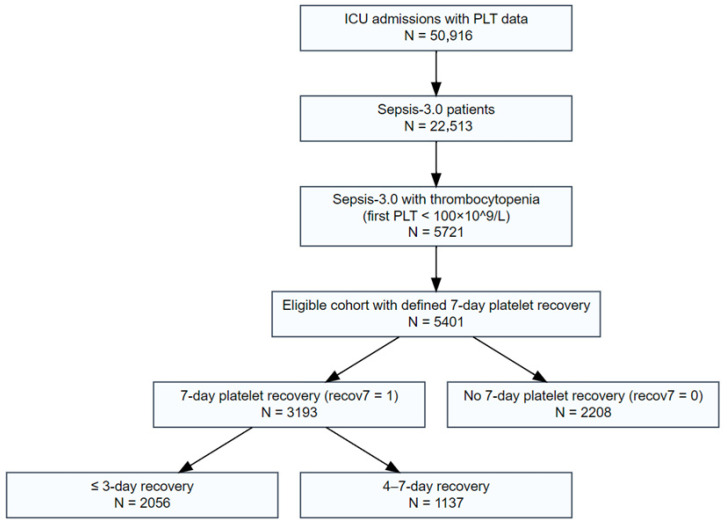
Patient Selection Flowchart. Derivation of the study cohort from the MIMIC-IV database. ICU admissions were screened for Sepsis-3.0 criteria, thrombocytopenia (platelet count < 100 × 10^9^/L), and adequate follow-up data to assess 7-day platelet recovery status. PLT denotes platelet count.

Cohort construction is illustrated in Figure 1. We identified all adult patients (≥18 years) with at least one ICU admission and available platelet measurements (*n* = 50,916). For patients with multiple admissions, only the first ICU stay was included. Sepsis was defined according to the Third International Consensus Definitions for Sepsis and Septic Shock (Sepsis-3)—documented or suspected infection accompanied by an acute increase of ≥2 points in the Sequential Organ Failure Assessment (SOFA) score [19]. Documented infection required a positive blood, urine, or respiratory culture within 48 h of ICU admission. Suspected infection was defined as initiation of antibiotics within 24 h. SOFA scores were calculated from the worst values during the first 24 h of ICU admission, consistent with Sepsis-3 guidelines [19].

Among patients with sepsis, thrombocytopenia was defined as an initial platelet count < 100 × 10^9^/L within the first 24 h of ICU admission (*n*= 5721). We excluded 320 patients with insufficient platelet monitoring to determine 7-day recovery status, yielding a final analytic cohort of 5401 patients.

## 3. Variables and Definitions

### 3.1. Exposure

The primary exposure was platelet recovery within 7 days after the onset of thrombocytopenia, defined as achieving and maintaining a platelet count ≥ 100 × 10^9^/L without subsequent decline. Patients with transient increases above this threshold were classified as non-recovered. Recovery was categorized as early (≤3 days), intermediate (4–7 days), or no recovery (>7 days or persistent platelet count < 100 × 10^9^/L). In analyses of the broader ICU cohort, patients who developed thrombocytopenia on the first ICU day were compared with those whose platelet counts remained ≥100 × 10^9^/L.

### 3.2. Outcomes

The primary outcome was 28-day all-cause mortality following ICU admission. Secondary outcomes included in-hospital mortality, ICU and hospital length of stay, survival time, and major complications: septic shock (per Sepsis-3 criteria), acute respiratory distress syndrome (ARDS), and acute kidney injury (AKI). ARDS and AKI were identified using standardized diagnostic codes, and severe AKI was additionally captured by initiation of continuous renal replacement therapy (CRRT). Vital status at 28 and 90 days was obtained from hospital records and linkage to the Social Security Death Index. Patients discharged alive were censored at discharge unless subsequent mortality data were available.

### 3.3. Covariates

Baseline covariates from the first 24 h of ICU admission included demographics (age, sex, race/ethnicity), body-mass index (BMI), comorbidities (hypertension, diabetes, hyperlipidemia, chronic kidney disease, cirrhosis, and immunosuppression), laboratory values (neutrophil and platelet counts), and severity of illness (SOFA score and Charlson Comorbidity Index). Comorbidities were identified using ICD-9/10 codes. Immunosuppression included solid-organ or stem-cell transplantation, active chemotherapy, chronic corticosteroid therapy (≥10 mg prednisone-equivalent daily for ≥3 months), or other immunosuppressive medications. ICU treatments administered before or during the platelet-recovery window included antibiotics, glucocorticoids, immunosuppressive agents, antihypertensives, nephrotoxic medications, heparin, other anticoagulants, antiplatelet agents, CRRT, and mechanical ventilation.

### 3.4. Statistical Analysis

Continuous variables were reported as medians with interquartile ranges (IQRs) and compared using the Mann–Whitney U test. Categorical variables were expressed as counts and percentages and compared using chi-square or Fisher's exact tests. All statistical tests were two-sided with a significance threshold of 0.05.

### 3.5. Propensity Score Methods

In the overall ICU cohort, we applied 1:1 nearest-neighbor propensity score matching without replacement using a caliper width of 0.2 SD of the logit of the propensity score. Propensity scores were estimated via logistic regression, including demographics, BMI, comorbidities, baseline neutrophil count, SOFA and Charlson scores, and ICU treatments. Covariate balance was assessed using standardized mean differences.

For the sepsis cohort and for comparisons of recovered versus non-recovered patients with thrombocytopenia, we used overlap weighting based on the same set of covariates. Effective sample sizes and standardized mean differences were used to assess weighting performance.

### 3.6. Regression Models

Risk ratios for binary outcomes were estimated using modified Poisson regression with robust variance. Length of stay and survival time were log-transformed and modeled with linear regression; estimates are presented as ratios on the original scale. Multivariable models adjusted for demographics, comorbidities, laboratory values, illness severity, and ICU treatments. Variables with substantial missingness or perfect collinearity were excluded from model fitting. All regression analyses were conducted in the predefined analytic cohort.

### 3.7. Additional Analyses

Among patients with sufficient longitudinal data (*n* = 2272), platelet trajectories over 14 days were plotted using LOESS smoothing (span = 0.75), with 95% confidence intervals obtained via bootstrap resampling. Kaplan–Meier survival analyses compared: (1) septic patients with vs. without thrombocytopenia, (2) thrombocytopenic patients stratified by 7-day platelet recovery status. Survival differences were evaluated using log-rank tests. Multivariable logistic regression, including all baseline demographic, clinical, laboratory, and treatment variables, was used to identify factors associated with 7-day platelet recovery. To assess the robustness of findings to potential time-dependent confounding and misclassification, additional landmark analyses were performed at days 3 and 7, with results presented in Supplementary Table S1.

### 3.8. Software

All analyses were conducted using R version 4.2.0. Propensity score matching used the MatchIt package; overlap weighting used WeightIt; robust variance estimation used sandwich; survival analyses used survival package; and figures were generated using ggplot2 and survminer.

## 4. Results

We screened 50,916 ICU admissions with platelet measurements, of which 22,513 (44.2%) met Sepsis-3.0 criteria. Thrombocytopenia developed within 24 h in 5721 patients (25.4%). After excluding 320 without adequate platelet follow-up, 5401 remained for analysis. Nearly 60% (*n* = 3193) achieved platelet recovery within 7 days, with roughly two-thirds of these (*n* = 2056, 38.1%) recovering by day 3 and the remainder (*n* = 1137, 21.1%) between days 4 and 7.

Baseline comparisons showed that patients who developed thrombocytopenia were older, had greater illness severity, and had more comorbid conditions than those who did not (Table 1). They had higher SOFA scores and greater prevalence of chronic kidney disease, cirrhosis, and immunosuppression, and they more frequently required mechanical ventilation, vasopressors, and continuous renal replacement therapy. Propensity-score matching yielded 5721 well-balanced pairs (standardized mean differences < 0.10; Table 2). Although crude 28-day mortality and ICU length of stay remained higher in the thrombocytopenia group, these differences were no longer present after adjustment (adjusted RR for 28-day mortality, 1.02; 95% CI, 0.96–1.09).

The findings were similar in the sepsis cohort. Thrombocytopenic patients again had greater baseline severity (Table 3), with unadjusted 28-day mortality of 32.1% compared with 21.5%. However, overlap-weighted analyses that accounted for illness severity and comorbidities showed no independent association between thrombocytopenia and mortality (adjusted RR, 1.04; 95% CI, 0.98–1.11).

**Figure 2:**
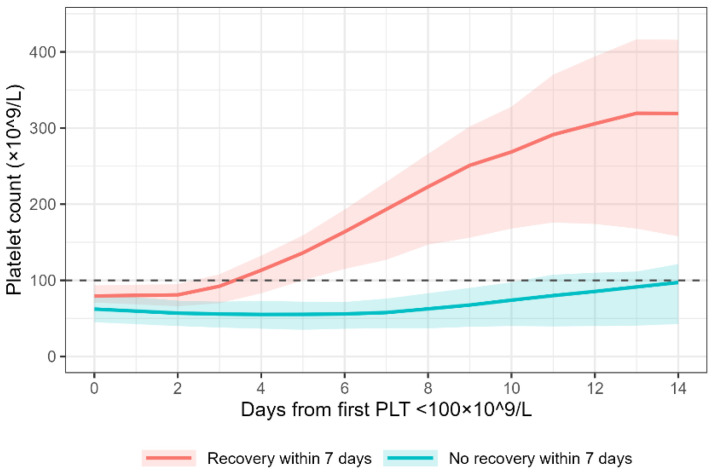
Platelet Count Trajectories by 7-Day Recovery Status. Smoothed platelet count trajectories over 14 days from thrombocytopenia onset, stratified by 7-day recovery status. Curves generated using LOESS smoothing; shaded areas represent 95% confidence intervals. Dashed line marks the recovery threshold (100 × 10^9^/L).

Among patients with thrombocytopenia, baseline characteristics differed substantially by recovery status (Table 4). Patients who recovered were younger, less severely ill, and had higher baseline platelet counts. Cirrhosis and CRRT use were less common, whereas antiplatelet therapy and heparin use were more frequent. Platelet trajectories in 2272 patients with sufficient longitudinal data (Figure 2) demonstrated these differences: patients with recovery exhibited a rapid and sustained rise in platelet counts, whereas those without recovery remained persistently thrombocytopenic through day 14.

**Figure 3:**
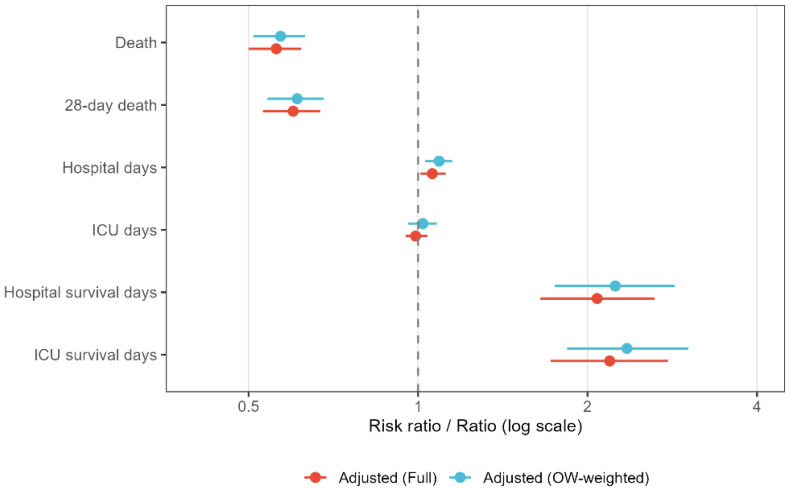
Adjusted Associations Between 7-Day Platelet Recovery and Clinical Outcomes in Septic Patients with Thrombocytopenia. Risk ratios for binary outcomes and ratios for continuous outcomes compare patients with 7-day platelet recovery with those without recovery, estimated using full multivariable models (red) and overlap-weighted models (blue). Horizontal lines denote 95% confidence intervals, and the vertical dashed line indicates the null value.

These associations were clinically meaningful. Patients achieving recovery within 7 days had markedly lower mortality—4.8% vs. 20.3% for in-hospital death and 21.4% vs. 43.5% for 28-day death (both *p* < 0.001). They also had longer survival and shorter ICU stays. These associations persisted after adjustment for demographics, comorbidities, severity of illness, laboratory values, and ICU treatments (Table 5). The adjusted risk ratio for in-hospital death was 0.56 (95% CI, 0.50–0.62) and for 28-day death was 0.60 (95% CI, 0.53–0.67). Overlap-weighted estimates were nearly identical, and survival time was more than doubled in the recovery group (adjusted ratio, 2.08; 95% CI, 1.65–2.63). Adjusted associations across outcomes are presented in Figure 3.

**Figure 4:**
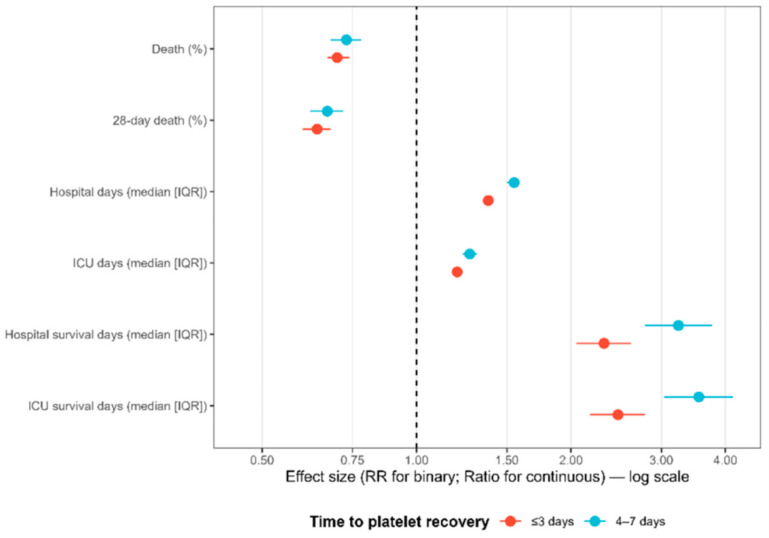
Adjusted Associations Between Time to Platelet Recovery and Clinical Outcomes in Septic Patients with Thrombocytopenia. Risk ratios for binary outcomes and ratios for continuous outcomes compare early (≤3 days, red) and intermediate (4–7 days, blue) recovery with no recovery, estimated from modified Poisson models adjusted for demographics, comorbidities, laboratory values, illness-severity scores, and ICU interventions. Horizontal lines indicate 95% confidence intervals, and the vertical dashed line marks the null value.

Recovery timing appeared less important than recovery itself. In exploratory timing-stratified analyses, both early (≤3 days) and intermediate (4–7 days) recovery showed similarly favorable associations with mortality compared with no recovery (Table 6), with overlapping confidence intervals. Confidence intervals overlapped, suggesting similar benefit regardless of recovery timing within the 7-day window. Ratios for survival time were unexpectedly higher in the intermediate group (3.24–3.55 vs. 2.32–2.47), likely reflecting survivor conditioning. These time-stratified effects are shown in Figure 4. Additional landmark analyses at days 3 and 7 yielded results consistent with the primary analysis, further supporting the robustness of the association between platelet recovery and mortality (Supplementary Table S1).

**Figure 5:**
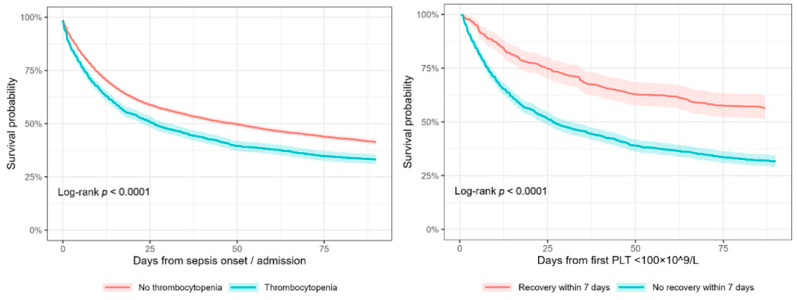
Kaplan–Meier Survival Curves by Thrombocytopenia and Recovery Status. The left panel shows 90-day survival among patients with and without thrombocytopenia from sepsis onset. The right panel shows 90-day survival among thrombocytopenic patients with and without platelet recovery within 7 days from the first platelet count < 100 × 10^9^/L. Shaded areas indicate 95% confidence intervals; log-rank *p* < 0.0001 for both comparisons.

Kaplan–Meier curves (Figure 5) provided further confirmation. Among all septic patients, thrombocytopenia was associated with worse unadjusted survival, supporting its role as a severity marker rather than an independent risk factor. Among thrombocytopenic patients, survival curves separated early and consistently favored those who achieved recovery.

Finally, predictors of 7-day recovery were evaluated (Table 7). Higher baseline platelet counts, antiplatelet therapy, and heparin use were associated with a greater likelihood of recovery. In contrast, higher SOFA and Charlson scores, cirrhosis, glucocorticoid use, and CRRT predicted a lower probability of recovery.

## 5. Discussion

This large retrospective cohort study of critically ill patients shows that platelet recovery after thrombocytopenia provides strong prognostic information in sepsis. Thrombocytopenia developed in approximately one-quarter of septic ICU patients within 24 h of admission, consistent with prior analyses of the MIMIC-IV and eICU databases [6,17,19]. These patients were older, had higher SOFA scores, and required more organ-support therapies, aligning with previous findings that thrombocytopenia reflects illness severity rather than acting as an independent cause of death [5,6,20]. Although crude mortality was higher among patients with thrombocytopenia, adjusted models demonstrated no independent association between thrombocytopenia and mortality, reinforcing its role as a marker of severity rather than a direct pathophysiologic contributor [21].

Among patients with thrombocytopenia, platelet recovery within seven days was strongly associated with improved survival, with nearly a twofold reduction in adjusted mortality and significantly longer survival time. Similar advantages have been observed in trajectory-based studies, in which patients with rising or recovering platelet patterns experienced substantially lower 28-day mortality [7,22,23]. These findings suggest that platelet restoration reflects broader physiologic stabilization—including recovery of hematopoiesis, inflammation, and endothelial function—capturing dynamic recovery signals that static platelet values cannot convey [5].

The timing findings should be interpreted as exploratory; within the prespecified 7-day window, the presence of recovery appeared more informative than the exact timing. Both early (≤3 days) and intermediate (4–7 days) recovery were associated with substantially lower mortality, with overlapping confidence intervals, consistent with earlier studies showing that recovery—regardless of timing—signals resolution of systemic inflammation and coagulopathy [14,15]. The slightly larger survival ratios observed with intermediate recovery likely reflect survivor conditioning rather than true biological differences.

Mechanistically, platelet restoration may represent the resolution of immune–coagulation dysregulation, reactivation of bone marrow function, and repair of endothelial injury. Persistent thrombocytopenia may instead indicate ongoing platelet consumption due to microvascular coagulation, immune-mediated clearance, or inflammation-driven suppression of thrombopoiesis [9,13,24]. Recent experimental and clinical studies also highlight the role of platelets in modulating host defense, releasing cytokines, and interacting with neutrophils and endothelial cells, suggesting that platelet recovery may serve as a surrogate for broader immune restoration [8,25].

Clinically, platelet recovery offers a readily available and dynamic marker of recovery trajectory, reflecting improvement in both hematologic status and overall physiologic stability. Incorporating platelet trajectories into sepsis risk assessment may improve individualized prognostication, consistent with emerging evidence that dynamic indicators—such as SOFA trajectories or the neutrophil–platelet ratio—outperform static measures for outcome prediction [22,26]. Future work combining these trajectories may refine sepsis phenotyping and therapeutic targeting.

This study has several strengths, including a large sample size, high-resolution longitudinal data, and robust methods using propensity-score matching and overlap weighting. As with other database studies, residual confounding, measurement variability, and the absence of coagulation or cytokine biomarkers limit causal inference [27]. Survivor bias may partially influence comparisons of early versus intermediate recovery, although sensitivity analyses demonstrated consistent findings. Validation in external ICU populations will be important.

## 6. Conclusions

In this cohort of 5401 septic patients with thrombocytopenia, platelet recovery within 7 days occurred in 59% of patients and was consistently associated with improved survival. After adjustment for clinical characteristics and illness severity, patients who achieved recovery had lower in-hospital mortality (adjusted RR, 0.56) and 28-day mortality (adjusted RR, 0.60) compared with those who did not. Exploratory analyses of recovery timing yielded similar associations across subgroups. These findings support platelet recovery as a useful prognostic marker in sepsis.

## Figures and Tables

**Table 1:** Baseline characteristics and ICU interventions before and after propensity-score matching.

Characteristics	Unmatched Cohort				Propensity-Matched Cohort			
	All	TP		All	TP		All	TP
N	50,916	4674	46,242		8782	4391	4391	
Age, years (%)								
18–39	4937 (9.7)	374 (8.0)	4563 (9.9)	<0.001	720 (8.2)	355 (8.1)	365 (8.3)	0.726
40–64	17,967 (35.3)	1999 (42.8)	15,968 (34.5)	<0.001	3429 (39.0)	1810 (41.2)	1619 (36.9)	<0.001
>65	28,012 (55.0)	2301 (49.2)	25,711 (55.6)	<0.001	4633 (52.8)	2226 (50.7)	2407 (54.8)	<0.001
Male (%)	28,439 (55.9)	2870 (61.4)	25,569 (55.3)	<0.001	5408 (61.6)	2693 (61.3)	2715 (61.8)	0.645
Race (%)								
AMERICAN	133 (0.3)	22 (0.5)	111 (0.2)	0.005	34 (0.4)	17 (0.4)	17 (0.4)	1.000
ASIAN	1496 (2.9)	169 (3.6)	1327 (2.9)	0.005	330 (3.8)	161 (3.7)	169 (3.8)	0.694
BLACK	4640 (9.1)	398 (8.5)	4242 (9.2)	0.143	743 (8.5)	372 (8.5)	371 (8.4)	1.000
HISPANIC	1735 (3.4)	187 (4.0)	1548 (3.3)	0.021	332 (3.8)	168 (3.8)	164 (3.7)	0.867
OTHER	2407 (4.7)	237 (5.1)	2170 (4.7)	0.261	410 (4.7)	217 (4.9)	193 (4.4)	0.245
UNKNOWN	6305 (12.4)	614 (13.1)	5691 (12.3)	0.106	1116 (12.7)	567 (12.9)	549 (12.5)	0.586
WHITE	34,200 (67.2)	3047 (65.2)	31,153 (67.4)	0.003	5817 (66.2)	2889 (65.8)	2928 (66.7)	0.391
BMI (median [IQR])	27.99 [27.99–27.99]	27.99 [27.20–27.99]	27.99 [27.99–27.99]	<0.001	27.99 [26.57–27.99]	27.99 [27.23–7.99]	27.99 [26.06–28.36]	0.030
Neutrophil count (median [IQR])	9.25 [6.20–13.14]	6.46 [3.37–10.43]	9.59 [6.62–13.39]	<0.001	8.43 [5.00–13.28]	6.43 [3.33–10.38]	10.83 [7.22–15.69]	<0.001
Comorbidities								
Type 2 Diabetes (%)	37,976 (74.6)	3569 (76.4)	34,407 (74.4)	0.004	6695 (76.2)	3339 (76.0)	3356 (76.4)	0.688
Hyperlipidemia (%)	32,619 (64.1)	3417 (73.1)	29,202 (63.2)	<0.001	6300 (71.7)	3157 (71.9)	3143 (71.6)	0.758
Chronic renal disease (%)	43,243 (84.9)	3890 (83.2)	39,353 (85.1)	<0.001	7266 (82.7)	3639 (82.9)	3627 (82.6)	0.756
Cirrhosis (%)	48,057 (94.4)	3309 (70.8)	44,748 (96.8)	<0.001	6735 (76.7)	3261 (74.3)	3474 (79.1)	<0.001
Immunosuppression (%)	49,649 (97.5)	4317 (92.4)	45,332 (98.0)	<0.001	8285 (94.3)	4079 (92.9)	4206 (95.8)	<0.001
SOFA (median [IQR])	3.0 [2–6]	7.0 [5–10]	3.0 [2–5]	<0.001	7.0 [5–10]	7.0 [5–10]	7.0 [5–10]	0.003
Charlson (median [IQR])	4.0 [2–7]	5.0 [3–7]	4.0 [2–7]	<0.001	5.0 [3–7]	5.0 [3–7]	5.0 [3–7]	0.132
Interventions								
Antibiotic (%)	36,724 (72.13)	32,691 (70.70)	4033 (86.29)	<0.001	878 (78.69)	396 (70.97)	482 (86.38)	<0.001
Glucocorticoids (%)	7078 (13.90)	6056 (13.10)	1022 (21.87)	<0.001	180 (16.13)	60 (10.75)	120 (21.51)	<0.001
Immunosuppressant drugs (%)	1267 (2.49)	910 (1.97)	357 (7.64)	<0.001	57 (5.11)	11 (1.97)	46 (8.24)	<0.001
Antihypertensive drugs (%)	31,277 (61.43)	28,233 (61.05)	3044 (65.13)	<0.001	698 (62.54)	341 (61.11)	357 (63.98)	0.322
Nephrotoxic drugs (%)	36,871 (72.42)	33,502 (72.45)	3369 (72.08)	0.590	790 (70.79)	411 (73.66)	379 (67.92)	0.035
Heparin (%)	40,136 (78.83)	36,931 (79.86)	3205 (68.57)	<0.001	834 (74.73)	443 (79.39)	391 (70.07)	<0.001
Anticoagulant drugs (%)	40,145 (78.85)	36,937 (79.88)	3208 (68.64)	<0.001	835 (74.82)	443 (79.39)	392 (70.25)	<0.001
Platelet aggregation drugs (%)	23,723 (46.59)	22,285 (48.19)	1438 (30.77)	<0.001	420 (37.64)	265 (47.49)	155 (27.78)	<0.001
Invasive ventilation (%)	17,652 (34.67)	15,801 (34.17)	1851 (39.60)	<0.001	419 (37.56)	205 (36.74)	214 (38.35)	0.578

Values are median [IQR] or number (%). TP denotes thrombocytopenia. Matched columns refer to propensity-score matched pairs. Comparisons used chi-square or Mann–Whitney U tests.

**Table 2:** Baseline Characteristics of the Sepsis-3.0 Cohort Before and After Propensity-Score Weighting.

Variables	Unmatched Cohort				Propensity-Matched Cohort (Weighted)			
	All	TP	No TP	*p*	All	TP	No TP	*p*
N	22,513	3041	19,270		1044	522	522	
N (ESS)	22,513	3041	19,270		484.7	522	157.8	
Age (%)				<0.001				0.635
18–39	1645 (7.31)	244 (8.02)	1374 (7.13)		63.4 (6.08)	35.0 (6.70)	28.4 (5.45)	
40–64	7696 (34.18)	1350 (44.39)	6290 (32.64)		469.6 (44.98)	227.0 (43.49)	242.6 (46.47)	
>65	13,172 (58.51)	1447 (47.58)	11,606 (60.23)		511.0 (48.94)	260.0 (49.81)	251.0 (48.08)	
Male (%)	13,019 (57.83)	1844 (60.64)	11,073 (57.46)	<0.001	644.8 (61.76)	307.0 (58.81)	337.8 (64.72)	0.127
Race (%)				<0.001				0.641
AMERICAN	59 (0.26)	14 (0.46)	43 (0.22)		2.8 (0.27)	2.0 (0.38)	0.8 (0.15)	
ASIAN	647 (2.87)	114 (3.75)	526 (2.73)		36.8 (3.52)	17.0 (3.26)	19.8 (3.79)	
BLACK	1813 (8.05)	235 (7.73)	1562 (8.11)		85.5 (8.19)	44.0 (8.43)	41.5 (7.96)	
HISPANIC	729 (3.24)	119 (3.91)	600 (3.11)		29.0 (2.78)	15.0 (2.87)	14.0 (2.68)	
OTHER	1056 (4.69)	157 (5.16)	887 (4.60)		48.6 (4.66)	28.0 (5.36)	20.6 (3.95)	
UNKNOWN	3121 (13.86)	434 (14.27)	2664 (13.82)		192.8 (18.47)	105.0 (20.11)	87.8 (16.82)	
WHITE	15,088 (67.02)	1968 (64.72)	12,988 (67.40)		648.5 (62.12)	311.0 (59.58)	337.5 (64.66)	
BMI (median [IQR])	28.12 [24.46–2.86]	27.56 [24.10–31.92]	28.24 [24.53–32.99]	<0.001	27.50 [23.73–32.55]	27.75 [24.34–32.63]	27.30 [23.17–32.3]	0.350
Neutrophil count (median [IQR])	10.06 [6.58–14.72]	6.67 [3.29–11.08]	10.72 [7.37–15.23]	<0.001	8.22 [4.85–11.79]	7.27 [3.63–11.33]	8.95 [6.07–12.18]	<0.001
Comorbidities								
Hypertension (%)	9681 (43.00)	1067 (35.09)	8540 (44.32)	<0.001	358.5 (34.34)	192.0 (36.78)	166.5 (31.90)	0.187
Type 2 Diabetes (%)	6318 (28.06)	723 (23.78)	5550 (28.80)	<0.001	231.1 (22.13)	119.0 (22.80)	112.1 (21.47)	0.668
Hyperlipidemia (%)	7982 (35.46)	781 (25.68)	7142 (37.06)	<0.001	300.8 (28.81)	155.0 (29.69)	145.8 (27.93)	0.589
Chronic renal disease (%)	4064 (18.05)	520 (17.10)	3508 (18.20)	0.141	203.0 (19.44)	104.0 (19.92)	99.0 (18.96)	0.722
Cirrhosis (%)	1948 (8.65)	986 (32.42)	952 (4.94)	<0.001	308.4 (29.54)	152.0 (29.12)	156.4 (29.96)	0.877
Immunosuppression (%)	5015 (22.28)	971 (31.93)	4003 (20.77)	<0.001	294.0 (28.16)	147.0 (28.16)	147.0 (28.15)	0.998
SOFA (median [IQR])	5.0 [3–8]	8.0 [6–11]	5.0 [3–7]	<0.001	10.0 [7–13]	10.0 [7–13]	10.0 [7–14]	0.433
Charlson (median [IQR])	5.0 [3–7]	5.0 [3–7]	5.0 [3–7]	<0.001	5.0 [3–7]	5.0 [3–7]	5.0 [3–7]	0.771
Intervention								
Antibiotic (%)	22,513 (100.00)	3041 (100.00)	19,270 (100.00)		1044 (100.00)	522 (100.00)	522 (100.00)	
Glucocorticoids (%)	3513 (15.60)	745 (24.50)	2756 (14.30)	<0.001	280.3 (26.85)	155.0 (29.69)	125.3 (24.01)	0.335
Immunosuppressant drugs (%)	758 (3.37)	255 (8.39)	502 (2.61)	<0.001	73.1 (7.01)	40.0 (7.66)	33.1 (6.35)	0.561
Antihypertensive drugs (%)	16,151 (71.74)	2039 (67.05)	14,027 (72.79)	<0.001	759.4 (72.74)	390.0 (74.71)	369.4 (70.77)	0.449
Heparin (%)	18,291 (81.25)	2153 (70.80)	15,979 (82.92)	<0.001	865.5 (82.90)	406.0 (77.78)	459.5 (88.03)	<0.001
Anticoagulant drugs (%)	18,926 (81.3)	2156 (70.90)	16,140 (82.90)	<0.001	867.5 (83.85)	405.0 (77.90)	460.5 (88.15)	<0.001
Platelet aggregation drugs (%)	11,195 (49.73)	885 (29.10)	10,248 (53.18)	<0.001	439.9 (42.14)	196.0 (37.55)	243.9 (46.72)	0.033
CRRT (%)	1196 (5.31)	347 (11.41)	848 (4.40)	<0.001	236.1 (22.62)	119.0 (22.80)	117.1 (22.43)	0.948
Invasive ventilation (%)	11,915 (52.92)	1450 (47.68)	10,406 (54.00)	<0.001	751.9 (72.02)	357.0 (68.39)	394.9 (75.64)	0.034

Values are median [IQR] or number (%). Comparisons used chi-square or Mann–Whitney U tests. Weighted counts reflect effective sample size.

**Table 3:** Associations Between Thrombocytopenia and Clinical Outcomes in the Sepsis-3.0 Cohort.

Outcomes	Unadjusted RR/Ratio (95% CI, *p*)	Adjusted RR/Ratio (Full) (95% CI, *p*)	Adjusted RR/Ratio (OW-Weighted) (95% CI, *p*)
Death	1.37 (1.32–1.43, *p* ≤ 0.001)	1.02 (0.98–1.07, *p* = 0.244)	1.05 (1.01–1.10, *p* = 0.027)
28-day death (%)	1.31 (1.25–1.36, *p* = 0.001)	0.98 (0.93–1.02, *p* = 0.276)	1.02 (0.97–1.07, *p* = 0.481)
Hospital days (median [IQR])	1.06 (1.03–1.09, *p* ≤ 0.001)	1.10 (1.06–1.13, *p* ≤ 0.001)	0.98 (0.95–1.01, *p* = 0.232)
ICU days (median [IQR])	0.98 (0.96–1.01, *p* = 0.281)	0.98 (0.95–1.00, *p* = 0.081)	0.86 (0.83–0.88, *p* ≤ 0.001)
Hospital survival days (median [IQR])	0.62 (0.55–0.69, *p* ≤ 0.001)	1.32 (1.18–1.47, *p* ≤ 0.001)	1.23 (1.08–1.39, *p* = 0.001)
ICU survival days (media [IQR])	0.58 (0.52–0.66, *p* ≤ 0.001)	1.30 (1.16–1.46, *p* ≤ 0.001)	1.22 (1.07–1.39, *p* = 0.002)

Risk ratios for binary outcomes and ratios for continuous outcomes were estimated with modified Poisson regression; continuous outcomes were modeled on log-transformed scales. Adjusted estimates controlled for demographics, comorbidities, laboratory values, illness-severity scores, and ICU interventions. OW-weighted estimates were obtained using overlap weighting based on propensity scores constructed from baseline covariates.

**Table 4:** Characteristics and Outcomes by 7-Day Platelet Recovery Status.

Characteristic	Unmatched Cohort				Propensity-Weighted Cohort			
	All	No Recovery	Recovery	*p*	All	No Recovery	Recovery	*p*
N	2272	1632	640		1043.3	403.3	640.0	
Age (%)								
18–39	188 (8.27)	100 (6.13)	88 (13.75)	<0.001	113.60 (10.89)	25.12 (6.23)	88.48 (13.83)	<0.001
40–64	1003 (44.15)	786 (48.16)	217 (33.91)	<0.001	411.22 (39.41)	192.76 (47.79)	218.47 (34.14)	<0.001
>65	1081 (47.58)	746 (45.71)	335 (52.34)	0.004	518.50 (49.70)	185.47 (45.98)	333.02 (52.04)	0.035
Male (%)	1402 (61.71)	1012 (62.01)	390 (60.94)	0.636	637.58 (61.11)	250.00 (61.98)	387.58 (60.56)	0.611
Race (%)								0.252
BMI (median [IQR])	27.82 [24.45–32.46]	28.08 [24.62–32.67]	27.23 [24.09–32.04]	0.042	27.65 [24.34–32.63]	28.43 [24.91–33.30]	27.15 [24.09–32.02]	0.012
Neutrophil count (median [IQR])	5.85 [1.57–10.26]	5.70 [1.49–10.07]	6.26 [3.39–11.51]	0.146	5.92 [1.85–10.51]	3.69 [1.09–8.39]	6.33 [3.49–11.53]	0.042
Platelet count (median [IQR])	72.3 [51.5–86.0]	65.50 [46.5–82.0]	82.0 [70.5–91.0]	<0.001	77.0 [60.0–89.0]	63.9 [44.7–81.0]	82.0 [70.6–91.0]	<0.001
Comorbidities								
Hypertension (%)	809 (35.61)	536 (32.84)	273 (42.66)	<0.001	405.69 (38.88)	139.15 (34.50)	266.54 (41.65)	0.010
Type 2 Diabetes (%)	575 (25.31)	434 (26.59)	141 (22.03)	0.025	234.26 (22.45)	98.22 (24.35)	136.05 (21.26)	0.192
Hyperlipidemia (%)	612 (26.94)	393 (24.08)	219 (34.22)	<0.001	316.90 (30.37)	98.95 (24.53)	217.95 (34.06)	<0.001
Chronic Renal disease (%)	424 (18.66)	332 (20.34)	92 (14.38)	0.001	171.59 (16.45)	78.78 (19.53)	92.81 (14.50)	0.019
Cirrhosis (%)	678 (29.84)	643 (39.40)	35 (5.47)	<0.001	192.53 (18.45)	158.33 (39.25)	34.19 (5.34)	<0.001
Immunosuppression (%)	706 (31.07)	579 (35.48)	127 (19.84)	<0.001	276.28 (26.48)	147.59 (36.59)	128.70 (20.11)	<0.001
SOFA (median [IQR])	9.0 [6–11]	9.0 [7–12]	7.0 [5–10]	<0.001	8.0 [6–11]	9.0 [6–12]	7.0 [5–10]	<0.001
Charlson (median [IQR])	5.0 [4–7]	6.0 [4–8]	4.0 [2–6]	<0.001	5.0 [3–7]	6.0 [4–8]	4.0 [2–6]	<0.001
Intervention								
Antibiotic (%)	2272 (100.00)	1632 (100.00)	640 (100.00)		1043.32 (100.0)	403.34 (100.00)	639.97 (100.00)	
Glucocorticoids (%)	626 (27.55)	553 (33.88)	73 (11.41)	<0.001	206.94 (19.83)	132.64 (32.89)	74.29 (11.61)	<0.001
Immunosuppressant drugs (%)	240 (10.56)	220 (13.48)	20 (3.12)	<0.001	67.41 (6.46)	48.12 (11.93)	19.29 (3.01)	<0.001
Antihypertensive drugs (%)	1836 (80.81)	1315 (80.58)	521 (81.41)	0.651	837.15 (80.24)	319.32 (79.17)	517.83 (80.91)	0.449
Heparin (%)	1908 (83.98)	1347 (82.54)	561 (87.66)	0.003	884.92 (84.82)	324.76 (80.52)	560.16 (87.53)	<0.001
Anticoagulant drugs (%)	1908 (83.98)	1347 (82.54)	561 (87.66)	0.003	884.92 (84.82)	324.76 (80.52)	560.16 (87.53)	<0.001
Platelet aggregation drugs (%)	837 (36.84)	505 (30.94)	332 (51.88)	<0.001	449.86 (43.12)	121.36 (30.09)	328.50 (51.33)	<0.001
CRRT (%)	331 (14.57)	296 (18.14)	35 (5.47)	<0.001	114.91 (11.01)	77.21 (19.14)	37.70 (5.89)	<0.001
Invasive ventilation (%)	1334 (58.71)	898 (55.02)	436 (68.12)	<0.001	652.88 (62.58)	217.38 (53.90)	435.50 (68.05)	<0.001
Outcomes								
Septic shock (%)	311 (13.69)	248 (15.20)	63 (9.84)	<0.001	133.79 (12.82)	68.95 (17.10)	64.83 (10.13)	<0.001
ARDS (%)	23 (1.01)	17 (1.04)	6 (0.94)	0.823	11.86 (1.14)	4.77 (1.18)	7.08 (1.11)	0.902
AKI (%)	1967 (86.58)	1430 (87.62)	537 (83.91)	0.019	887.19 (85.04)	352.27 (87.34)	534.92 (83.58)	0.062
Hospital days (median [IQR])	14.64 [9.77–23.89]	16.01 [10.22–26.81]	12.20 [8.86–17.89]	<0.001	13.07 [9.16–20.99]	15.79 [10.26–25.35]	12.17 [8.79–18.13]	<0.001
ICU days (median [IQR])	5.14 [2.68–10.21]	5.27 [2.71–10.87]	4.94 [2.58–8.77]	0.014	5.04 [2.72–9.64]	5.23 [2.78–11.30]	4.98 [2.57–8.66]	0.015
Death (%)	363 (15.98)	332 (20.34)	31 (4.84)	<0.001	114.72 (11.00)	83.13 (20.61)	31.59 (4.94)	<0.001
28-day death (%)	847 (37.28)	710 (43.50)	137 (21.41)	<0.001	320.09 (30.68)	184.21 (45.67)	135.88 (21.23)	<0.001
Hospital survival days(median [IQR])	51.72 [17.52–236.24]	46.35 [16.38–214.91]	110.31 [34.08–371.92]	<0.001	70.15 [18.99–262.64]	47.94 [15.95–210.36]	111.51 [34.00–364.93]	<0.001
ICU survival days (median [IQR])	47.60 [15.55–229.22]	41.93 [14.75–211.95]	106.33 [34.01–371.92]	<0.001	66.19 [16.92–262.58]	41.42 [14.35–199.81]	110.71 [33.92–364.93]	<0.001

Values are median [IQR] or number (%). Recovery was defined as a sustained platelet count ≥ 100 × 109/L within 7 days. Weighted counts were derived from overlap weighting based on baseline covariates. Comparisons used chi-square or Mann–Whitney U tests.

**Table 5:** Associations Between 7-Day Platelet Recovery and Clinical Outcomes in Septic Patients with Thrombocytopenia.

Outcomes	Unadjusted (95% CI, *p*)	Adjusted (95% CI, *p*)	OW-Weighted (95%CI, *p*)
Death	0.44 (0.40–0.49, *p* < 0.001)	0.56 (0.50–0.62, *p* < 0.001)	0.57 (0.51–0.63, *p* < 0.001)
28-day death (%)	0.49 (0.44–0.54, *p* < 0.001)	0.60 (0.53–0.67, *p* < 0.001)	0.61 (0.54–0.68, *p* < 0.001)
Hospital days (median [IQR])	0.94 (0.89–0.99, *p* = 0.030)	1.06 (1.01–1.12, *p* = 0.030)	1.09 (1.03–1.15, *p* = 0.004)
ICU days (median [IQR])	0.92 (0.87–0.97, *p* = 0.002)	0.99 (0.95–1.04, *p* = 0.759)	1.02 (0.96–1.08, *p* = 0.501)
Hospital survival days (median [IQR])	2.80 (2.22–3.53, *p* < 0.001)	2.08 (1.65–2.63, *p* < 0.001)	2.24 (1.75–2.86, *p* < 0.001)
ICU survival days (media [IQR])	3.05 (2.41–3.87, *p* < 0.001)	2.19 (1.72–2.78, *p* < 0.001)	2.35 (1.84–3.02, *p* < 0.001)

Values are median [IQR] or number (%). Estimates for binary outcomes used modified Poisson regression; continuous outcomes were modeled on log-transformed scales. Adjusted models included demographics, comorbidities, laboratory values, illness-severity scores, and ICU interventions. Overlap-weighted estimates were derived from propensity scores based on baseline covariates.

**Table 6:** Clinical Outcomes According to Time to Platelet Recovery in Septic Patients with Thrombocytopenia.

Outcome	Time to Recovery	Event Rate/Median [IQR]	Unadjusted RR (95% CI)	*p*	Adjusted RR/Ratio (95% CI)	*p*
Death (%)	≤3 days	28.8% (916/3184)	0.88 (0.83–0.93)	<0.001	0.70 (0.67–0.74)	<0.001
	4–7 days	33.2% (534/1608)	1.02 (0.95–1.09)	0.592	0.73 (0.68–0.78)	<0.001
28-day death (%)	≤3 days	24.6% (782/3184)	0.80 (0.75–0.85)	<0.001	0.64 (0.60–0.68)	<0.001
	4–7 days	28.2% (453/1608)	0.92 (0.85–0.99)	0.029	0.67 (0.62–0.72)	<0.001
Hospital days (median [IQR])	≤3 days	8.9 [6.1–15.2]	—	—	1.38 (1.35–1.41)	<0.001
	4–7 days	11.3 [7.2–18.8]	—	—	1.55 (1.50–1.59)	<0.001
ICU days (median [IQR])	≤3 days	2.8 [1.5–5.3]	—	—	1.20 (1.18–1.23)	<0.001
	4–7 days	3.2 [1.7–7.3]	—	—	1.27 (1.23–1.31)	<0.001
Hospital survival days (median [IQR])	≤3 days	88.5 [21.8–371.0]	—	—	2.32 (2.05–2.62)	<0.001
	4–7 days	110.3 [26.3–369.2]	—	—	3.24 (2.79–3.77)	<0.001
ICU survival days (media [IQR])	≤3 days	87.6 [19.2–370.9]	—	—	2.47 (2.18–2.79)	<0.001
	4–7 days	108.5 [24.3–368.6]	—	—	3.55 (3.04–4.14)	<0.001

Risk ratios compare early (≤3 days) and intermediate (4–7 days) platelet recovery with no recovery. Adjusted estimates were derived from modified Poisson regression including demographics, comorbidities, laboratory values, illness-severity scores, and ICU interventions. Continuous outcomes were modeled on log-transformed scales, and only adjusted ratios are reported.

**Table 7:** Factors Associated with 7-Day Platelet Recovery in Septic Patients with Thrombocytopenia.

Risk Factor	Category/Unit	Unadjusted OR (95% CI, *p*)	Adjusted OR (95% CI, *p*)
Age	18–39 (ref)	1.00	1.00
	40–64	—	0.94 (0.45–1.93, *p* = 0.864)
	≥65	—	0.85 (0.38–1.87, *p* = 0.690)
Hypertension	No (ref)	1.00	1.00
	Yes	1.40 (1.25–1.57, *p* < 0.001)	1.08 (0.76–1.54, *p* = 0.674)
Hyperlipidemia	No (ref)	1.00	1.00
	Yes	1.83 (1.61–2.07, *p* < 0.001)	1.04 (0.72–1.49, *p* = 0.833)
Chronic renal disease	No (ref)	1.00	1.00
	Yes	1.02 (0.88–1.18, *p* = 0.796)	2.44 (1.50–4.02, *p* < 0.001)
Cirrhosis	No (ref)	1.00	1.00
	1	0.25 (0.22–0.28, *p* < 0.001)	0.47 (0.31–0.71, *p* < 0.001)
Immunosuppression	No (ref)	1.00	1.00
	1	0.58 (0.51–0.65, *p* < 0.001)	1.19 (0.79–1.82, *p* = 0.412)
BMI (per IQR)	per IQR	0.98 (0.91–1.06, *p* = 0.575)	1.15 (0.98–1.36, *p* = 0.092)
Neutrophils (per IQR)	per IQR	1.17 (1.05–1.31, *p* = 0.007)	0.99 (0.85–1.17, *p* = 0.916)
Baseline platelets (per IQR)	per IQR	2.12 (1.96–2.29, *p* < 0.001)	1.63 (1.34–2.00, *p* < 0.001)
SOFA (per IQR)	per IQR	0.58 (0.54–0.63, *p* < 0.001)	0.63 (0.49–0.81, *p* < 0.001)
Charlson (per IQR)	per IQR	0.61 (0.57–0.66, *p* < 0.001)	0.48 (0.35–0.65, *p* < 0.001)
Glucocorticoids drugs	No (ref)	1.00	1.00
	1	0.45 (0.40–0.52, *p* < 0.001)	0.62 (0.42–0.91, *p* = 0.015)
Immunosuppressant drugs	No (ref)	1.00	1.00
	1	0.51 (0.41–0.62, *p* < 0.001)	0.73 (0.36–1.51, *p* = 0.399)
Antihypertensive drugs	No (ref)	1.00	1.00
	1	1.94 (1.71–2.21, *p* < 0.001)	1.51 (1.00–2.28, *p* = 0.050)
Heparin	No (ref)	1.00	1.00
	1	1.73 (1.53–1.97, *p* < 0.001)	1.81 (1.17–2.81, *p* = 0.008)
Platelet aggregation drugs	No (ref)	1.00	1.00
	1	3.42 (3.04–3.85, *p* < 0.001)	2.69 (1.89–3.83, *p* < 0.001)
CRRT	No (ref)	1.00	1.00
	1	0.50 (0.42–0.59, *p* < 0.001)	0.38 (0.25–0.59, *p* < 0.001)
Invasive ventilation	No (ref)	1.00	1.00
	1	1.88 (1.68–2.10, *p* < 0.001)	1.41 (0.98–2.03, *p* = 0.065)

Odds ratios reflect the likelihood of achieving 7-day platelet recovery, comparing factor levels or per-IQR increments for continuous variables. Adjusted estimates from multivariable logistic regression, including all listed covariates.

## Data Availability

The MIMIC-IV database is publicly available at https://mimic.mit.edu/ (accessed on 10 December 2025). The analysis code is available from the corresponding author upon reasonable request.

## Supplementary Materials

The following supporting information can be downloaded at: https://www.mdpi.com/article/10.3390/jcm15020884/s1, Table S1: Landmark Analyses of Platelet Recovery and Mortality in Septic Patients with Thrombocytopenia.
