# Supplementary material for: Platelet Recovery and Mortality in Septic Patients with Thrombocytopenia: A Propensity Score-Matched Analysis of the MIMIC-IV Database
Source: J Clin Med. 2026 Jan 21;15(2):884. doi: 10.3390/jcm15020884 (PMC12842087; doi:10.3390/jcm15020884)
Supplement: Supplementary file 1 [file jcm-15-00884-s001.zip › jcm-4065131-supplementary.pdf]

Table S1. Landmark Analyses of Platelet Recovery and Mortality in Septic Patients with Thrombocytopenia

Landmark Time	Exposure Definition	Adjusted RR	95% CI
Day 3	Platelet recovery before Day 3	1.05	1.03–1.07
Day 7	Platelet recovery before Day 7	1.08	1.05–1.11
Day 3	No platelet recovery before Day 3	0.95	0.93–0.97
Day 7	No platelet recovery before Day 7	0.93	0.90–0.95

Risk ratios for mortality were estimated using modified Poisson regression with robust variance. At each landmark time (day 3 or day 7), only patients who remained alive and under follow-up were included in the risk set. Platelet recovery status prior to the landmark was treated as a fixed exposure, and associations with subsequent mortality were evaluated from the landmark forward. Adjusted models included demographics, comorbidities, laboratory values, illness-severity scores, and ICU interventions.
